# GSK-3α Is a Novel Target of CREB and CREB-GSK-3α Signaling Participates in Cell Viability in Lung Cancer

**DOI:** 10.1371/journal.pone.0153075

**Published:** 2016-04-06

**Authors:** Sin-Aye Park, Jong Woo Lee, Roy S. Herbst, Ja Seok Koo

**Affiliations:** 1 Section of Medical Oncology, Department of Internal Medicine, Yale Comprehensive Cancer Center, Yale School of Medicine, New Haven, Connecticut, 06520, United States of America; 2 Developmental Therapeutics, Translational Research Program, Yale Comprehensive Cancer Center, New Haven, Connecticut, 06520, United States of America; Florida International University, UNITED STATES

## Abstract

Overexpression or activation of cyclic AMP-response element-binding protein (CREB) has been known to be involved in several human malignancies, including lung cancer. Genes regulated by CREB have been reported to suppress apoptosis, induce cell proliferation, inflammation, and tumor metastasis. However, the critical target genes of CREB in lung cancer have not been well understood. Here, we identified GSK-3α as one of the CREB target genes which is critical for the viability of lung cancer cells. The CREB knockdown significantly reduced the expression of GSK-3α and the direct binding of CREB on the promoter of *GSK3A* was identified. Kaplan-Meier analysis with a public database showed a prognostic significance of aberrant GSK-3α expression in lung cancer. Inhibition of GSK-3α suppressed cell viability, colony formation, and tumor growth. For the first time, we demonstrated that GSK-3α is regulated by CREB in lung cancer and is required for the cell viability. These findings implicate CREB-GSK-3α axis as a novel therapeutic target for lung cancer treatment.

## Introduction

The transcription factor cyclic AMP-response element-binding protein (CREB) regulates diverse cellular processes which include cell differentiation, proliferation, survival, glucose metabolism, immune regulation, and synaptic plasticity associated with memory [1–7]. Previously, we showed that CREB is critical for the regulation of mucous differentiation of normal human tracheobronchial epithelial (NHTBE) cells [8]. In addition, the decreased survival duration was significantly associated with overexpression of CREB or activated CREB (p-CREB) in never smokers with non-small cell lung cancer (NSCLC)[9] and the knockdown of CREB suppresses the viability of lung cancer cells [10]. Several serine-threonine kinases can activate CREB and p-CREB induces the expression of multiple cAMP response element-containing genes, those of which play important roles in the function of CREB. Several approaches for identifying CREB target genes have been reported [11–14], but distinct target genes of CREB in lung cancer remain largely unknown.

GSK-3, which has two isoforms of GSK-3α and GSK-3β, is a serine/threonine protein kinase that is involved in cell-cycle progression, differentiation, and apoptosis. GSK-3 is constitutively active in resting cells and it phosphorylates and inhibits oncogenic signaling such as β-catenin/WNT pathway [15–21]. Although GSK-3 has been studied as a tumor suppressor [22–24], there is increasing evidence that GSK-3 plays an oncogenic role in various human cancers. Most studies have focused on the role of total GSK-3 or GSK-3β [25–27], but recent studies implicated the oncogenic role of GSK-3α in acute myeloid leukemia (AML) [28], prostate cancer [29], and pancreatic cancer [30]. Interestingly, CREB overexpression or its increased activity has been linked to the progression of those human cancers [31–36]. In particular, CREB functions as a proto-oncogene in AML [31, 37] and GSK-3α is also a critical target for AML therapy [28]. Recently, GSK-3α and GSK-3β have been reported to be new kinase targets of tivantinib, which is a potent selective inhibitor of the receptor tyrosine kinase c-MET, in lung cancer cells. Tivantinib showed higher potency for GSK-3α more than for GSK-3β and the inhibition of GSK-3α or GSK-3β expression caused apoptosis in lung cancer cells [38].

Here, we first identified that GSK-3α, not GSK-3β, is regulated by CREB in lung cancer cells. Furthermore, we examined that there is a positive correlation between high GSK-3α expression and shorter survival of lung cancer patients. Knockdown of GSK-3α attenuates cell viability, colony formation, and tumor growth. Together, these findings implicates that GSK-3α is a critical target gene of CREB and CREB-GSK-3α signaling is a potential therapeutic target for lung cancer.

## Materials and Methods

### Cell culture

Human lung cancer cell lines (H1993, H1437, H1734, and A549) were obtained from the American Type Culture Collection. Lung cancer cells were cultured in RPMI-1640 medium (Invitrogen), supplemented with 10% (volume/volume) heat-inactivated fetal bovine/serum (FBS; Sigma Aldrich), 2 mM L-glutamine, 100 U/ml of penicillin G sodium and 100 μg/ml of streptomycin sulfate (Invitrogen). Normal human tracheobronchial epithelial cells (NHTBE) were obtained from the Lonza Walkersville, Inc. and cultured in BEGM^™^ with several supplements. All cells have been passaged directly from original low-passage stocks and were used before passage 30. The cells were also tested within the last three months for correct morphology by microscope and to detect mycoplasma contamination using a MycoAlert mycoplasma detection kit (Lonza Walkersville, Inc.). All cells were cultured at 37°C in humidified atmosphere of 95% air and 5% CO_2_.

### Antibodies/Chemicals

Monoclonal anti-β-actin antibody (A2228) was purchased from Sigma Aldrich. Rabbit polyclonal antibody against GSK-3α (ab28833) was purchased from Abcam. Forskolin (3828), anti-CREB (9197), anti-p-CREB (9198), anti-cyclin A2 (4656), anti-cyclin B1 (4138), anti-cyclin E2 (4132), and GSK-3β (12456) were obtained from Cell Signaling Technology. Rabbit monoclonal cyclin D1 antibody (2261–1) was purchased from Epitomics.

### Knockdown/ Overexpression of Genes

Silencer CREB siRNA and GSK-3α siRNAs were purchased from Thermo scientific or Invitrogen; CREB siRNA (109994, Invitrogen), GSK-3α siRNA-1 (L-003009-00-0005, ON-TARGETplus SMARTpool, ThermoScientific), and GSK-3α siRNA-2 (145366, Invitrogen). BLOCK-it Fluorescent Oligo (Invitrogen) was used as a control. Each siRNA was transfected using Lipofectamine RNAiMAX (Invitrogen). Also, cells were infected with lentiviral shScrambled or shRNAs targeting CREB or GSK-3α with 8 μg/ml polybrene and the infected cells were selected with puromycin. The sequences of shRNAs are listed in the S1 Table (available online). For CREB overexpression, H1437 and A549 cells were transfected with plasmid DNA of pCMV-empty or pCMV-CREB (Clontech Laboratories, Inc.) using Lipofectamine 2000 (Invitrogen).

### Cell Viability

Cell viability was evaluated by MTT assay. After cells were transfected with siRNAs for 72 h, the cells were incubated with MTT (final concentration 0.5 mg/ml) for 4 h at 37°C incubator. Following MTT incubation, 150 μl of 100% DMSO was added to dissolve the crystals. Viable cells were counted by reading the absorbance at 570 nm using a microplate reader SpectraMax (Molecular Devices).

### Colony Formation Assay

At 24 h after transfection by the indicated siRNAs, 2 x 10^3^ cells were transferred in the 6-well plates and allowed to grow for 7–14 days. The medium was removed, fixed with 10% formalin for 15 min, and followed by staining with crystal violet to visualize the colonies.

### Quantitative Real-time PCR

Total RNA was purified from cells using an RNeasy Mini Kit (Qiagen). Reverse transcription of total RNA was performed using the M-MLV reverse transcriptase (Promega). Quantitative PCR (qPCR) was performed using *SYBR* Green PCR Core Reagents (Applied Biosystems) and iCycler thermal cycler (Bio-Rad Laboratories). Primer sequences are listed in the S1 Table (available online).

### Western Blot Analysis

Standard SDS-PAGE and western blotting procedures were used to analyze the expression of various proteins. Whole cell lysates from each of the lung cancer cell lines tested were prepared using SDS lysis buffer (50 mM Tris-HCl, pH 6.8, 2% SDS, 10% glycerol, and 0.02% bromophenol blue) containing protease inhibitors and phosphatase. All proteins were visualized using a horseradish peroxidase-conjugated secondary antibody and Amersham ECL^™^ Western Blotting Detection Reagents (GE Healthcare Life Sciences). Intensity of individual bands was quantified using ImageJ densitometry software and expressed relative to actin signal, as a measure of protein relative abundance in the different samples.

### Chromatin Immunoprecipitation (ChIP)

The SimpleChIP Enzymatic kit (Cell Signaling) was used as described by the manufacturer. PCR was performed with primers specific for the indicated promoter regions and the reactions were performed in triplicate and 1% of the total input sample was used as a control. Primer sequences are listed in the S1 Table (available online).

### Immunostaining

For immunofluorescence, detection of primary antibodies was done using fluorescent conjugates of Alexa Fluor^®^ 488 antibody (Invitrogen) along with ProLong^®^ Gold Antifade Reagent with DAPI (Invitrogen). Before staining of fixed paraffin-embedded tissues, we followed the standard protocol which included steps such as deparaffinization, antigen retrieval, and permeabilization.

### Flow Cytometry

For cell cycle flow cytometry, the cells were fixed in 70% ethanol and stained with propidium iodine staining (BD Pharmingen) for DNA content. Apoptosis was measured using the FITC Annexin V Apoptosis Detection Kit (BD Pharmingen) following the manufacturer’s instruction.

### *In Vivo* Studies

All procedures were approved by the Institutional Animal Care and Use Committee (IACUC) at Yale University and conformed to the legal mandates and federal guidelines for the care and maintenance of laboratory animals (protocol #: 2012–11464). Female J:NU nude mice were obtained from Jackson Laboratory and used when 6–7 weeks old. H1993 cells were pre-treated with 20 nM of control siRNA or GSK-3α siRNA for 24 h, followed by transplantation (2 x 10^6^ cells/flank, xenograft n = 7/ group) into the flank of mice. Also, H1993-shScrambled, H1993-shGSK3A#2, or H1993-shGSK3A#4 cells were inoculated at dorsal flanks (6 x 10^5^ cells/flank; shScrambled n = 9, shGSK3A#2 n = 4, and shGSK3A#4 n = 5). All xenografts were transplanted in both the right and left dorsal flanks of mice. Tumor volume was measured with digital calipers and calculated by the formula 0.52 x length x width^2^. Mice were sacrificed at the end of the study by being placed in a carbon dioxide chamber.

### Statistical Methods

The quantitation results are presented as means ± standard deviation (SD). The statistical significance of differences among the groups was determined by Student’s *t*-test, with a *P* value below 0.01 considered to be statistically significant. The Kaplan-Meier method was used to analyze univariate survival, and comparisons of the survival distributions among groups were performed using the log-rank test.

## Results

### GSK-3α is regulated by CREB in lung cancer cells

**Fig 1:**
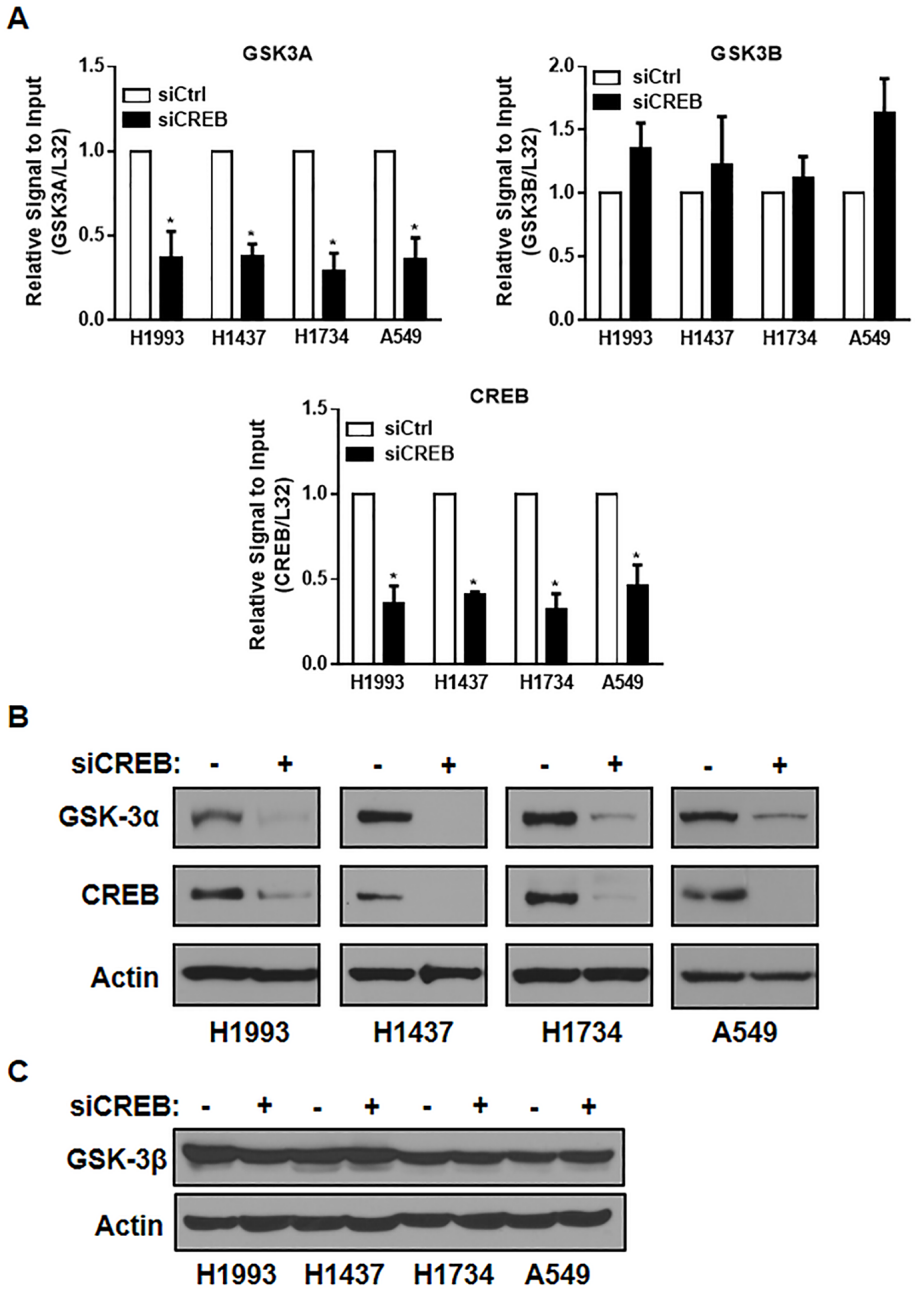
CREB inhibition suppresses the expression of GSK-3α. (A) Effect of CREB knockdown on the mRNA level of *GSK3A*, *GSK3B*, and *CREB*. Each of the indicated cells were transfected with control siRNA or CREB siRNA (40 nM, each) for 48 h, followed by qPCR analysis. All values in the graphs represent mean ± SD of three independent experiments. Two-sided *t-*test. *, *P* < 0.01. (B-C) Effect of CREB knockdown on the protein levels of GSK-3α (B) and GSK-3β (C). Each of the indicated cells were transfected with control siRNA or CREB siRNA (40 nM, each) for 72 h, followed by western blot analysis.

To investigate the critical CREB target genes in lung cancer, we performed qPCR analysis using specific primers against a subset of genes related to cell survival, proliferation, and viability in CREB knockdown cells. We found that the mRNA level of GSK-3α, not the level of GSK-3β, was significantly downregulated by CREB siRNA in all lung cancer cell lines we tested (H1993, H1437, H1734, and A549) (Fig 1A). In addition, the protein expression of GSK-3α was dramatically suppressed by CREB knockdown in all four lung cancer cell lines we tested (Fig 1B) but the protein level of GSK-3β was not changed by CREB knockdown (Fig 1C).

**Fig 2:**
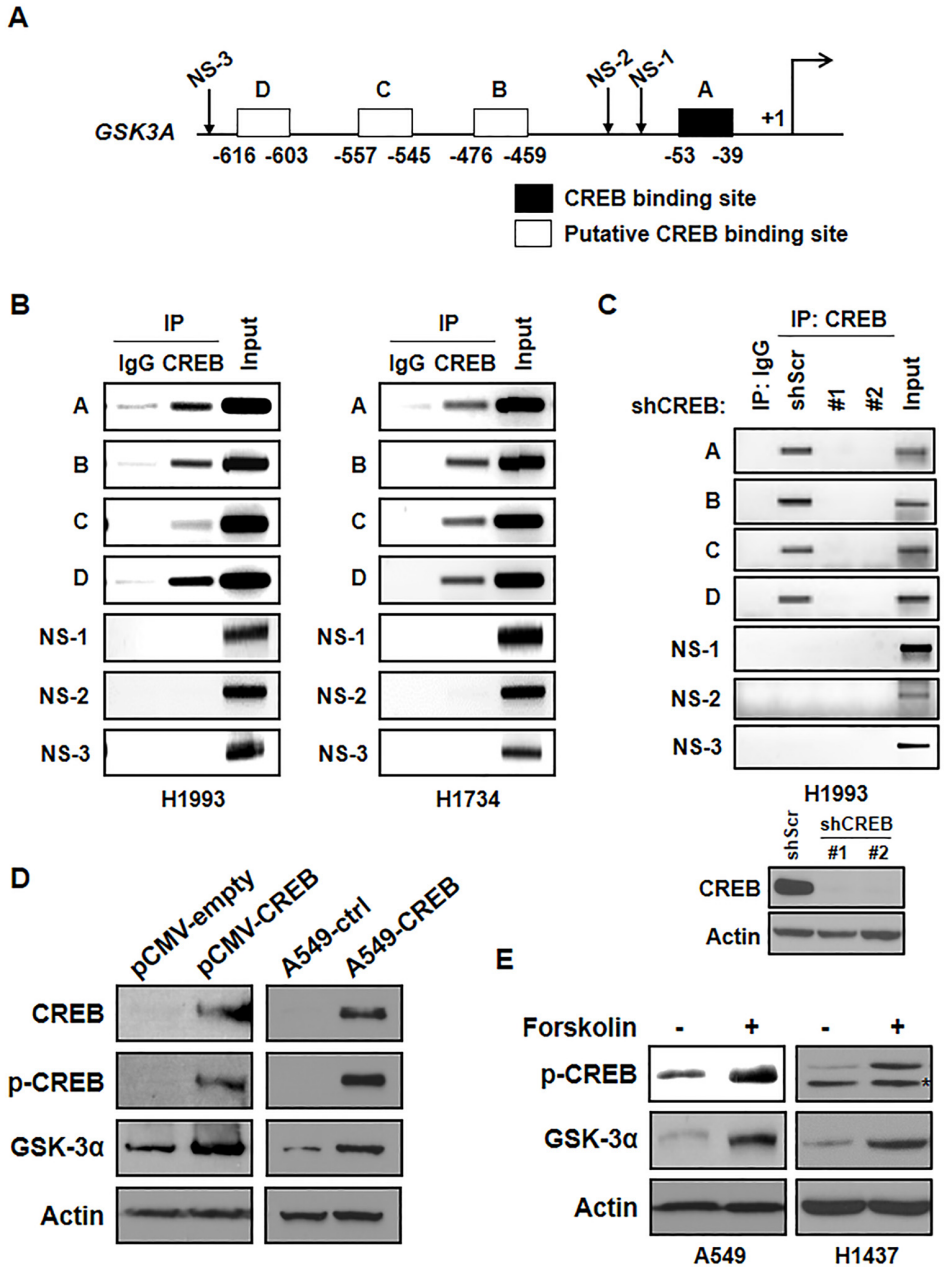
CREB binds to the promoter of GSK-3α and regulates the expression of GSK-3α. (A) Schematic diagram showing the positions of CREB binding elements located in the gene promoter of human *GSK3A* (TFSEARCH). A-D: the specific regions for primers which cover CREB binding elements, A: -39 to -53, B: -459 to -476, C: -545 to -557, D: -603 to -616, NS-1,-2, and -3: the regions for primers which include non-specific binding elements NS-1: -126 to -230, NS-2: -183 to -378, and NS-3: -1214 to -1356. Primer sequences are in the S1 Table (available online). (B) Direct binding of CREB on the *GSK3A* promoter. A ChIP assay was done with chromatins prepared from H1993 and H1734 cells. The binding of CREB to the *GSK3A* promoter was detected by visualization of the PCR product. The single bands detected in input samples indicate the specificity of the PCR primers. (C) Direct binding of CREB on the *GSK3A* promoter in CREB-knockdown cells. The protein level of CREB in each stable cell line was confirmed by western blot analysis. (D) Effect of CREB overexpression on the expression of GSK-3α. H1437 cells were transfected with the same amount of pCMV-empty or pCMV-CREB expression vector and the expression of CREB, p-CREB, and GSK-3α was examined by western blot analysis (left). A549-control (A549-ctrl) or A549-CREB cells were transfected with pCMV vectors and selected by puromycin (1 μg/ml). The effect of CREB on the expression of GSK-3α was also confirmed (right). (E) Effect of forskolin on the induction of the level of p-CREB and GSK-3α. A549 and H1437 cells were treated with forskolin (10 μM, 30 min) and the expression of each protein was examined by western blot analysis.

We noticed that the *GSK3A* promoter contained several putative CREB binding sites as determined by TFSEARCH (http://www.cbrc.jp/research/db/TFSEARCH.html) (Fig 2A). To examine whether CREB binds to the human *GSK3A* promoter, we performed ChIP assay by using immunoprecipitation of CREB antibody and IgG as a negative control. Not only primer set A which covers the CREB consensus binding site (-39 to -53), but also three other primer sets (B: -459 to -476, C: -545 to -557, and D: -603 to -616) showed the binding of CREB on the *GSK3A* promoter. However, non-specific regions (NS-1, NS-2, and NS-3) of the promoter did not show any binding of CREB (Fig 2B). In addition, the knockdown of CREB markedly suppressed the association of CREB with the *GSK3A* promoter (Fig 2C). Consistent with previous data which showed CREB knockdown suppressed the expression of GSK-3α, overexpression of CREB strongly induced GSK-3α expression at the protein level after transient or stable overexpression of CREB (Fig 2D). In fact, the expressions of p-CREB and GSK-3α were increased by forskolin, which activates the enzyme adenylyl cyclase and increases intracellular levels of cyclic AMP (Fig 2E). Taken together, we suggest that CREB is a potential upstream regulator of GSK-3α in lung cancer cells.

### GSK-3α is a poor prognosis factor in lung cancer

**Fig 3:**
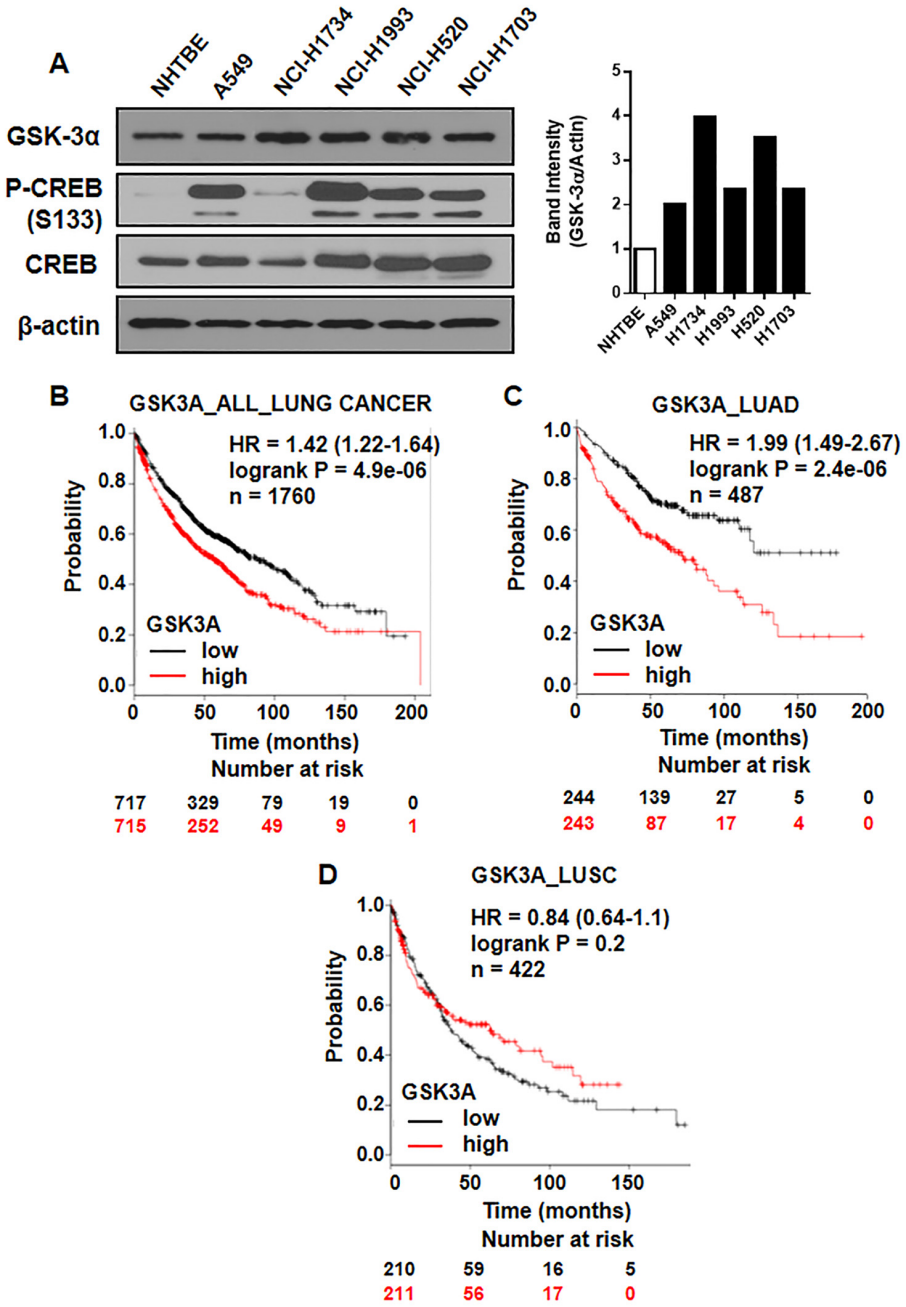
GSK-3α overexpression indicates poor prognosis of lung cancer. (A) Protein levels of GSK-3α, p-CREB, and CREB in multiple lung cancer cell lines compared with NHTBE cells. (B-D) Kaplan-Meier analysis of overall survival by low or high *GSK3A* (*GSK3A* probe set 202210_x_at) expression in (B) 1760 lung cancer patients, (C) 487 lung adenocarcinoma patients, and (D) 422 lung squamous cell carcinoma with adjuvant treatment. Overall survival analysis of the patients was performed by using Cox proportional hazard models and follow-up data for the indicated period.

There is evidence that GSK-3β is overexpressed in lung cancer. The overexpression of GSK-3β serves as an independent marker of poor prognosis for NSCLC and its inhibition suppresses cell proliferation in NSCLC cells [39]. However, the role of GSK-3α in lung cancer still need more investigation. Here, we found that GSK-3α is overexpressed in multiple lung cancer cell lines compared with NHTBE cells (Fig 3A).

To further assess whether our finding of *GSK3A* overexpression is relevant to human lung cancer, we used publically available Kaplan-Meier plotter (http://kmplot.com/analysis), which is consist of 1760 lung cancer patients who receive chemo/radiotherapy based on the databases (CARRAY: n = 504; GSE14814: n = 90; GSE19188: n = 156; GSE29013: n = 55; GSE31210: n = 246; GSE3141: n = 111; GSE37745: n = 196; GSE4573: n = 131; GSE8894: n = 138; and TCGA: n = 133). To determine whether *GSK3A* mRNA (202210_x_at) abundance in tumors was associated with overall survival, we performed on overall survival analysis of the patients using Cox proportional hazard models and follow-up data for 200 months after surgery. Overexpression of *GSK3A* mRNA levels was associated with poor overall survival of lung cancer patients (harzard ratio (HR) = 1.42, logrank P = 4.9e-06) (Fig 3B). Interestingly, *GSK3A* mRNA levels were more strongly associated with poor overall survival of lung cancer patients with adenocarcinoma (HR = 1.99, logrank P = 2.4e-06) (Fig 3C). However, positive *GSK3A* expression was not significantly correlated with shorter survival time of the patients with squamous cell carcinoma histology type (Fig 3D). We also found that overexpression of *CREB* mRNA level was associated with poor overall survival of lung cancer patients with very similar pattern for *GSK3A* mRNA (S2 Fig). Our results on clinical tumor samples demonstrate that aberrant activation of GSK-3α is associated with human mortality of lung cancer patients, especially with lung adenocarcinoma.

### Inhibition of GSK-3α suppresses the viability of lung cancer cells

**Fig 4:**
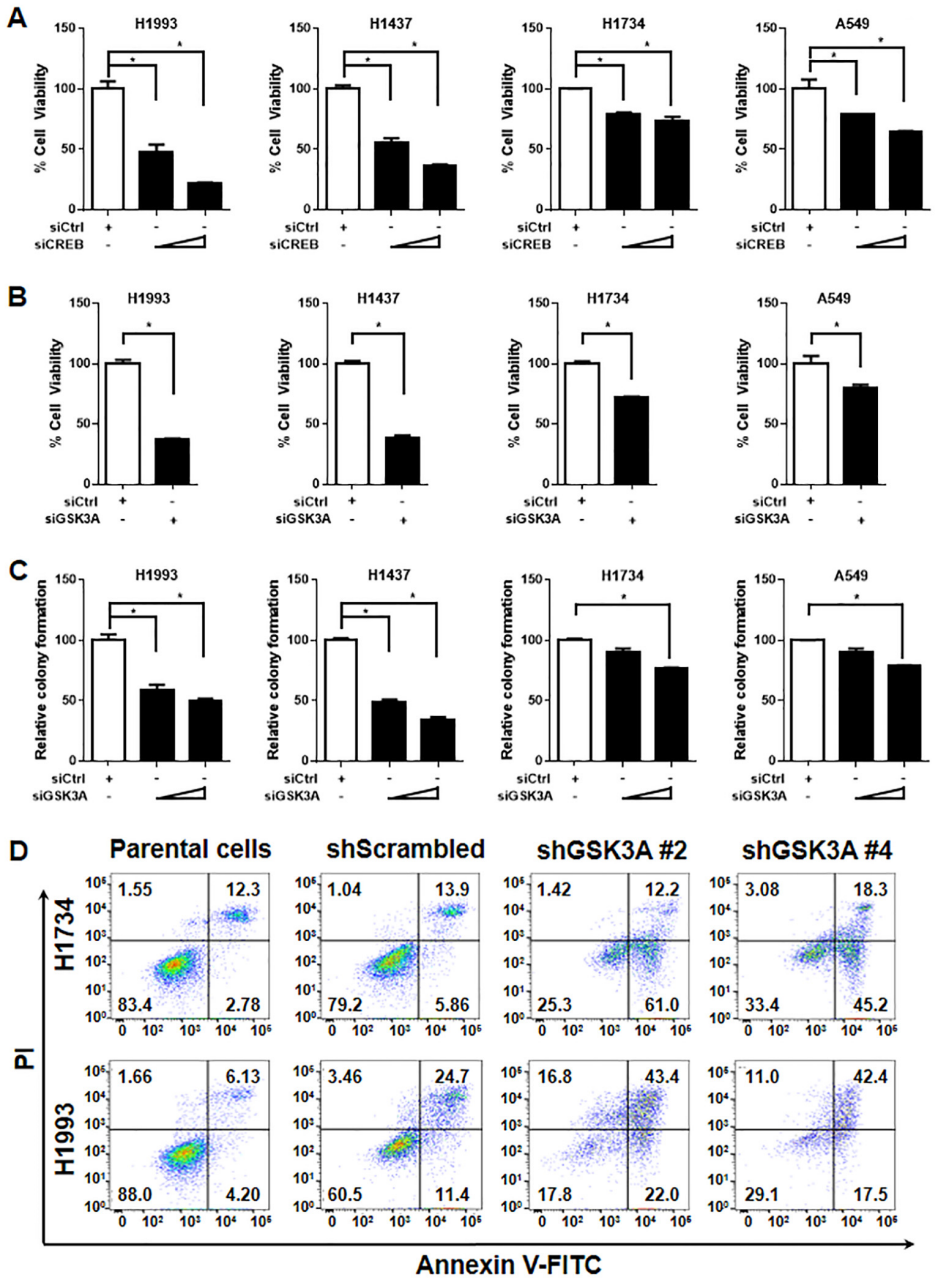
GSK-3α knockdown suppresses lung cancer cell viability. (A) Effect of CREB knockdown on the cell viability. Lung cancer cell lines (H1993, H1437, H1734, and A549) were transiently transfected with control siRNA (50 nM), or CREB siRNA (10, 50 nM) for 72 h, followed by MTT assay. All values in the graphs represent mean ± SD of three independent experiments. Two-sided *t*-test. *, *P* < 0.01. (B) Effect of GSK-3α knockdown on the cell viability. The cells were transiently transfected with control siRNA, or GSK-3α siRNA (40 nM, each) for 72 h and the quantitative data was followed by MTT assay. All values in the graphs represent mean ± SD of three independent experiments. Two-sided *t-*test. *, *P* < 0.01. (C) Effect of GSK-3α knockdown on colony formation. One day after transfection of control siRNA (40 nM), or GSK-3α siRNA (10, 40 nM), the cells were seeded again in 6-wells with low density (2 x 10^3^/ well) and incubated for 7–14 days. Mean ± SD in three independent experiments. Two-sided *t-*test. *, *P* < 0.01. (D) Effect of GSK-3α knockdown on the cell death. H1734 and H1993 cells were infected with lentiviral expressing shRNA targeting GSK-3α (two sequences; shGSK3A#2 and shGSK3A#4) with 8 μg/ml polybrene. After 48 h infection, cells were selected with 0.5 μg/ml puromycin for 3 days. The selected cells were performed by staining of annexin V and PI to analysis apoptotic cell death by LSRII.

To determine the effect of GSK-3α, which is a CREB target gene, we confirmed the effect of CREB knockdown on the cell viability in multiple lung cancer cell lines. Consistent with our previous reports [10], CREB inhibition suppressed the viability of lung cancer cells (Fig 4A). Next, knockdown of GSK-3α with its specific siRNA resulted in a decrease in the viability of all lung cancer cell lines we tested (Fig 4B). In addition, knockdown of GSK-3α led to reduced colony formation of the cells (Fig 4C). Consequently, GSK-3α knockdown significantly suppressed the cell viability of KRAS-WT lung cancer cell lines (H1993 and H1437), compared to KRAS-mutant lung cancer cell lines (H1734 and A549). We further examined the effect of GSK-3α knockdown on the cell death using FACS analysis. As presented in Fig 4D, the cells which were expressing shGSK3A showed the increased cell death compared with each control cell line. The validation of siRNAs or shRNAs on the expression of GSK-3α was performed by qPCR or western blot analysis in multiple lung cancer cell lines (S1 Fig). These results suggest that GSK-3α positively regulates the viability of lung cancer cells.

### CREB-GSK-3α signaling is critical for the regulation of cyclins

**Fig 5:**
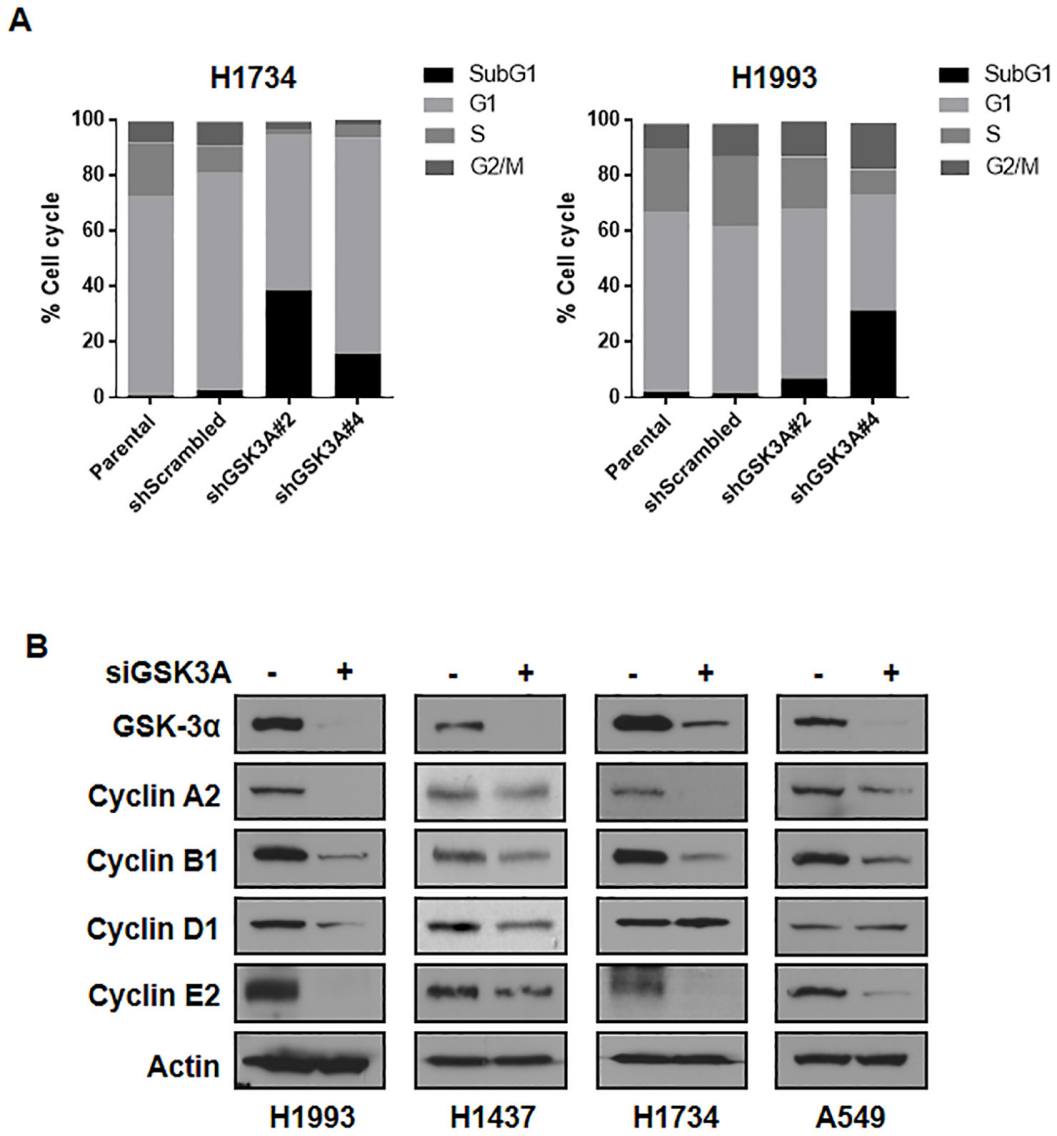
Effects of GSK-3α knockdown on the expression of cyclins. (A) Effect of GSK-3α knockdown on the cell cycle. Indicated cells were starved in serum-free RPMI medium for 24 h and replenished with RPMI supplemented with 10% FBS for another 24 h. Harvested cells were stained with PI to analysis cell cycle by LSRII. (B) Effect of GSK-3α knockdown on the gene expressions related to cell viability or cell cycle including cyclin A2, cyclin B1, cyclin D1, and cyclin E2. The lung cancer cells were transiently transfected with control siRNA, or GSK-3α siRNA (40 nM, each) for 48 h and were followed by western blot analysis.

To gain insight into the role of GSK-3α in lung cancer cell viability, we first examined whether GSK-3α regulates cell cycle using FACS analysis. Interestingly, GSK-3α knockdown suppressed S phase of the cell cycle, which might contribute the reduced cell viability of lung cancer cells (Fig 5A). As shown in Fig 5B, the protein expression of several cyclins including cyclin A2, cyclin B1, cyclin D1, and cyclin E2 was markedly suppressed by GSK-3α knockdown in lung cancer cell lines. In addition, these results were consistent with previous reports that CREB can regulate the expression of cyclins [12, 31, 40, 41]. Taken together, these results suggested that GSK-3α might function positively in the viability of lung cancer cells by regulating the expression of cyclins.

### GSK-3α is critical for tumor growth

**Fig 6:**
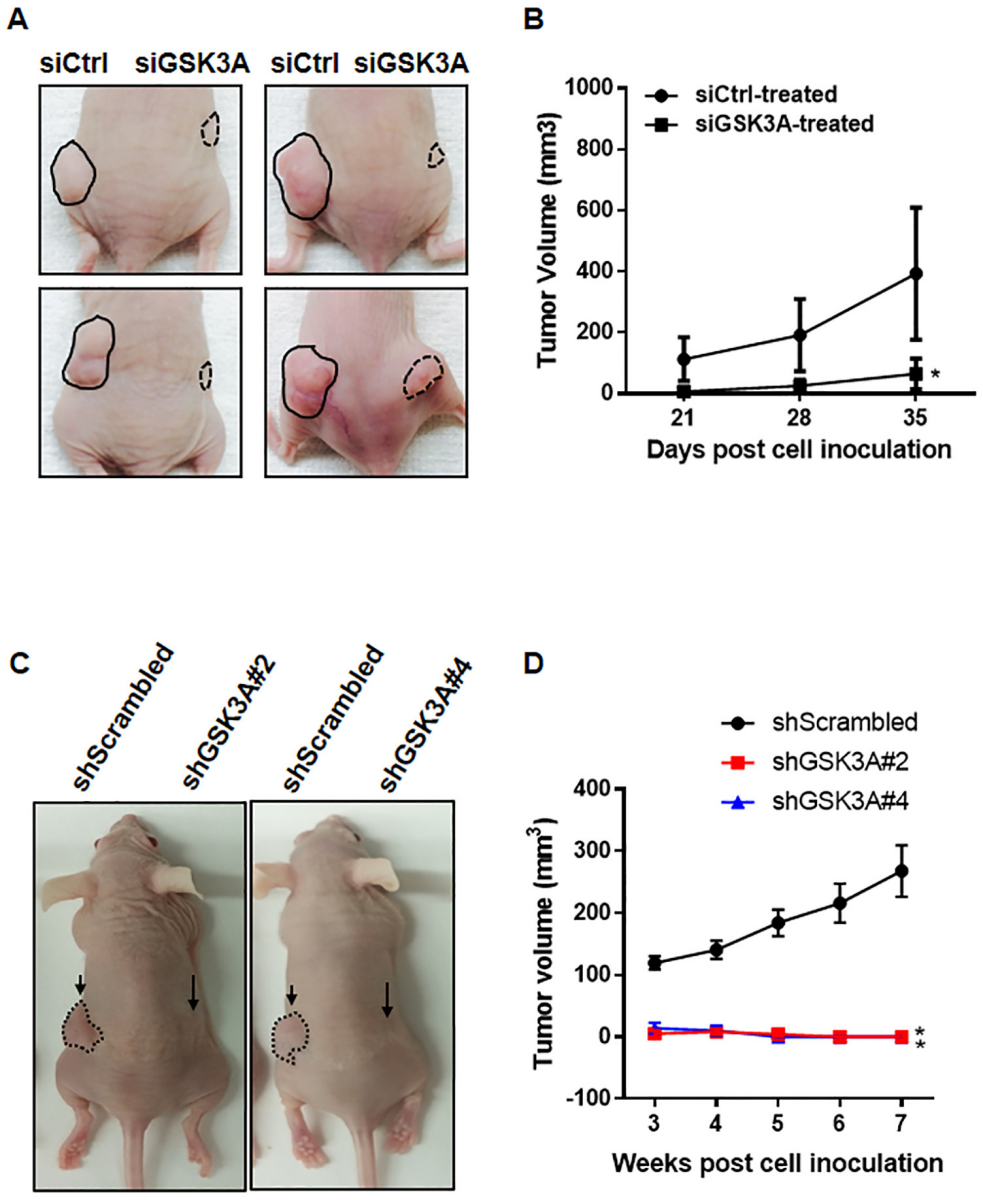
Effects of GSK-3α-deficient cells on the capability of tumor growth *in vivo*. (A) Representative images of xenografts derived from control siRNA or GSK-3α siRNA-treated H1993 cells. After 24 h siRNA transfection (20 nM, each), the cells were inoculated subcutaneously into the right and left side dorsal flanks of female nude mice (xenograft n = 7/group). (B) Tumor volume of xenografts derived from control or GSK-3α-deficient H1993 cells were evaluated at each time point as indicated. Tumor volume was measured with digital calipers and calculated by the formula 0.52 x length x width^2^. Two-sided *t*-test. *, *P* < 0.01. (C) Representative images of xenografts derived from H1993-shScrambled, shGSK3A#2, or shGSK3A#4 cells. The cells were inoculated subcutaneously into dorsal flanks of nude mice (left: shScrambled cells, right: shGSK3A cells) and tumor volume was measured over the indicated time points (shScrambled n = 9, shGSK3A#2 n = 4, and shGSK3A#4 n = 5). Two-sided *t*-test. *, *P <* 0.05.

We next addressed the role GSK-3α in tumor growth *in vivo* using subcutaneous injections of control or GSK-3α-depleted cells. The growth of tumors was monitored over five weeks after the cells were explanted in nude mice. Representative photographs of mice at the end of five weeks showed that the development of tumors derived from the GSK-3α-depleted cells was markedly suppressed compared with tumors derived from control cells (Fig 6A). Consistent with our hypothesis and data, GSK-3α knockdown resulted in a significant suppression in tumor growth and tumor volume (Fig 6B). We confirmed the effect of GSK-3α depletion on the expression of GSK-3α or cyclin B1 in the tissues derived from xenografts (S5 Fig). Repeatedly, we noticed that the complete deletion of *GSK3A* in the cells could not develop the tumors in nude mice (Fig 6C and 6D). Overall, these data clearly demonstrate that the decreased levels of GSK-3α impair lung cancer tumor growth *in vivo*.

## Discussion

In this study, we first identified GSK-3α as a novel target of CREB in lung cancer cells. Our study reveals oncogenic roles of GSK-3α as a CREB target gene and as a novel prognostic biomarker in lung cancer. GSK-3α has been shown to be a therapeutic target in multiple human cancers including AML, pancreatic cancer, and prostate cancer. However, the role of GSK-3α in lung cancer still largely remains unknown. Here, our results show that decreased levels of GSK-3α impair the viability of lung cancer cells *in vitro* and *in vivo*. GSK-3α is overexpressed in multiple lung cancer cell lines and lung tumor tissues. Also, the aberrant overexpression of GSK-3α was associated with a shorter survival time especially in patients with lung adenocarcinoma. More importantly, CREB regulates the expression of GSK-3α but not GSK-3β, suggesting that there is a specific arm of CREB-GSK-3α signaling in lung cancer.

The activity of CREB is regulated by multiple phosphorylation mechanisms and the phosphorylation of CREB at serine-133 is required for recruitment of the co-activator CREB-binding protein (CBP)/p300 and its transcriptional activity. In fact, GSK-3 has been known as a repressor of CREB activity. CREB DNA binding activity is inhibited by GSK-3β overexpression and increased by lithium or sodium valproate which are GSK-3 inhibitors in human neuroblastoma cells [42]. Moreover, GSK-3β has been reported to repress multiple CREB-target genes [43, 44]. Conversely, recent study has been reported that GSK-3 promotes the association of CREB and its co-activators with MEIS1, a homeobox (HOX) DNA-binding cofactor, to induce HOX-mediated transcription and transformation in MLL leukemias [45]. Although the functional consequence of CREB activity by GSK-3 is not still clear, our study strongly suggests that CREB positively regulates GSK-3α, not GSK-3β, in lung cancer cells, thus providing a new concept of CREB-GSK-3α signaling.

Although the overexpression of GSK-3β and its function as a tumor promoter in lung cancer has been demonstrated [39], the role of GSK-3α in lung cancer remains elusive. In our current study, we found that GSK-3α is overexpressed in lung cancer cell lines compared to normal bronchial epithelial cells. Knockdown of GSK-3α in lung cancer cells causes suppression of cell proliferation and also an induction of substantial apoptosis. These results indicate that GSK-3α also plays a critical role in the growth of lung cancer cells.

The mechanism of GSK-3α as a tumor promoter in lung cancer is virtually unknown. It has been shown that GSK-3α promotes oncogenic KRAS function via IKK-NF-κB activity in pancreatic cancer. The authors suggested GSK-3α as a key downstream effector of mutant KRAS to regulate NF-κB signaling pathways [30]. Interestingly, our study showed that the GSK-3α knockdown highly suppressed the cell viability of KRAS-WT lung cancer cell lines, H1993 and H1437 cells, compared to KRAS-mutant lung cancer cell lines, H1734 (G13C) and A549 (G12S) cells. Although it remains unclear whether KRAS status is directly related to the role of GSK-3α in lung cancer cells, we suggest that the function of GSK-3α in KRAS signaling might be regulated in a cell-type specific manner.

To better understand the role of GSK-3α and examine the critical genes affected by GSK-3α in lung cancer, we initially screened a subset of NF-κB target genes such as *MYC*, *WT1*, *BIRC2*, *IL-9*, *HMOX1*, and *TERT*, which were regulated by a pan-GSK-3 inhibitor AR-014418 in pancreatic cancer [30]. Our data was not fully consistent with the previous study, but we observed a significant decrease in the mRNA level of TERT by GSK-3α knockdown and little difference in the other NF-κB targets the analyzed (EDN1, CYP19A1, HMOX1, WT1, and BIRC2) (S3 Fig). Additionally, we found that the expression of several cyclins which are known as CREB target gens was markedly downregulated by GSK-3α knockdown in multiple lung cancer cell lines. Though our study did not directly identify how cyclins are regulated by GSK-3α, these results strongly support the novel role of GSK-3α as an active regulator in the viability of lung cancer cells.

Theoretically, the inhibition of GSK-3 can lead to β-catenin stabilization and hyperactivation of Wnt/β-catenin signaling [46–49]. However, Doble et al. showed that both isoforms of GSK-3 need to be inhibited for β-catenin stabilization. GSK-3α/β double-knockout mouse embryonic stem cell (ESC) displayed hyperactivated Wnt/β-catenin signaling [50]. Consistent with this report, there is increasing evidence that the single loss of either GSK-3α or GSK-3β isoform does not lead to β-catenin stabilization [28, 39]. Our data also demonstrated that inhibition of GSK-3α does not induce the level of β-catenin and AXIN2, an Axin-related protein which plays an important role in Wnt/β-catenin signaling pathway (S4 Fig). These results support that GSK-3α might function independent of the β-catenin stabilization in lung cancer but further analysis needs to be completed to fully understand the roles of GSK-3α and GSK-3β in β -catenin stabilization in lung cancer.

The observations that GSK-3α expression plays a causal role in survival of lung cancer patients, suggest GSK-3α could be useful as a prognostic biomarker in lung cancer, especially lung adenocarcinoma. Further studies examining the mechanisms for targeting CREB-GSK-3α pathway in lung cancer will be essential for determining and developing the GSK-3α specific inhibitors. In conclusion, we propose that GSK-3α is a promising therapeutic target as a novel target gene of CREB to diagnose tumor and develop the therapy, with relevance for lung cancer.

## Supporting Information

S1 Fig The validation of siRNAs and shRNAs on expression of GSK-3α in lung cancer cells. (TIF)

S2 Fig Kaplan-Meier analysis of overall survival by low or high CREB expression in lung cancer patients. (TIF)

S3 Fig The effect of GSK-3α on the expression of NF-κB target genes. (TIF)

S4 Fig The effect of GSK-3α on the expression of β-catenin or AXIN2. (TIF)

S5 Fig The level of GSK-3α or cyclin B1 in tissues derived from xenografts. (TIF)

S1 Table Primer sequences for qPCR or ChIP assay and sequences of shRNAs. (DOCX)
