# Supplementary figures and images for: GSK-3α Is a Novel Target of CREB and CREB-GSK-3α Signaling Participates in Cell Viability in Lung Cancer
Source: PLoS One. 2016 Apr 6;11(4):e0153075. doi: 10.1371/journal.pone.0153075 (PMC4822949; doi:10.1371/journal.pone.0153075)

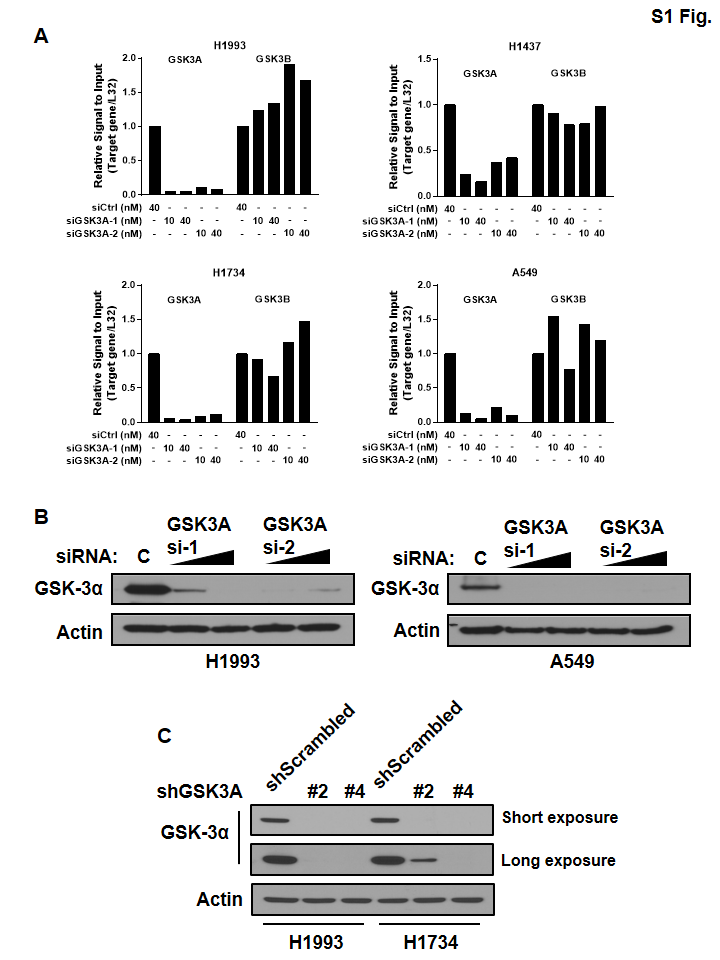

Supplement: S1 Fig — (TIF) [file pone.0153075.s001.tif]

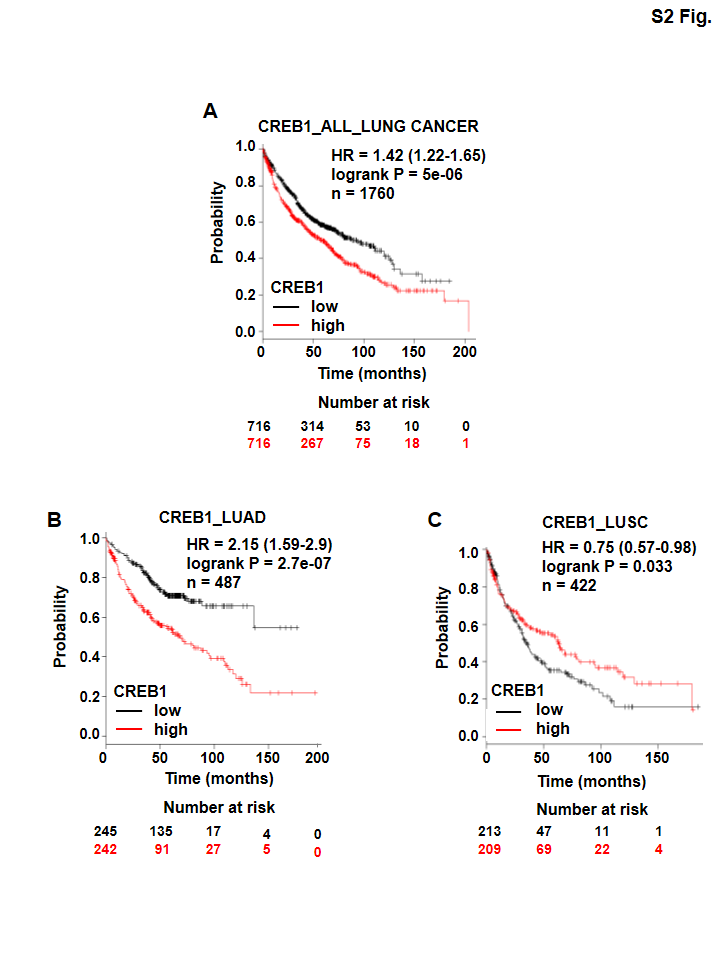

Supplement: S2 Fig — (TIF) [file pone.0153075.s002.tif]

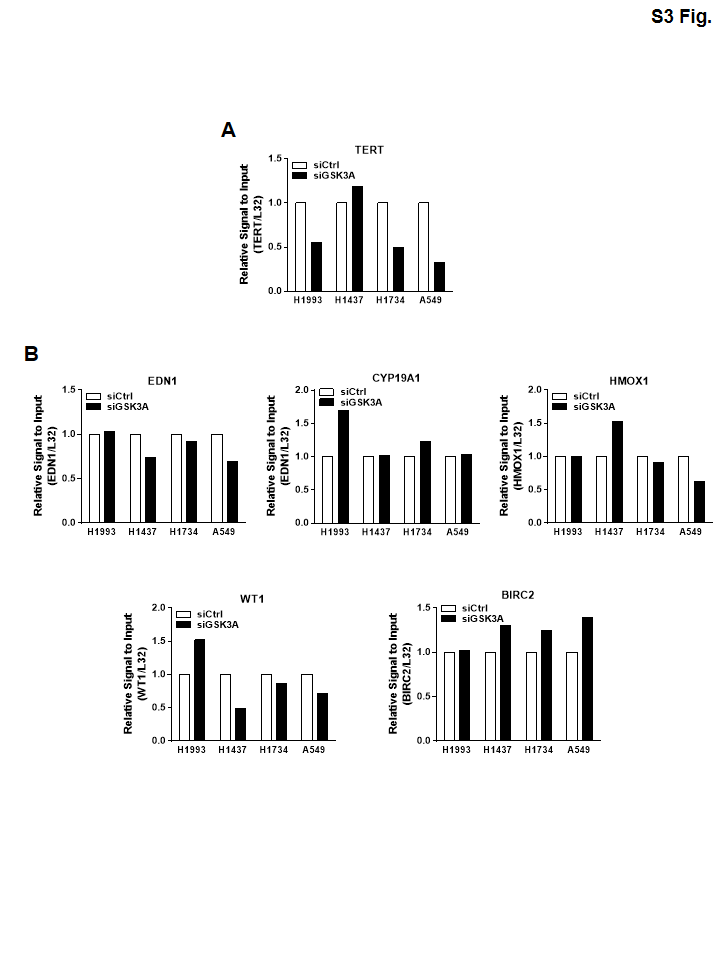

Supplement: S3 Fig — (TIF) [file pone.0153075.s003.tif]

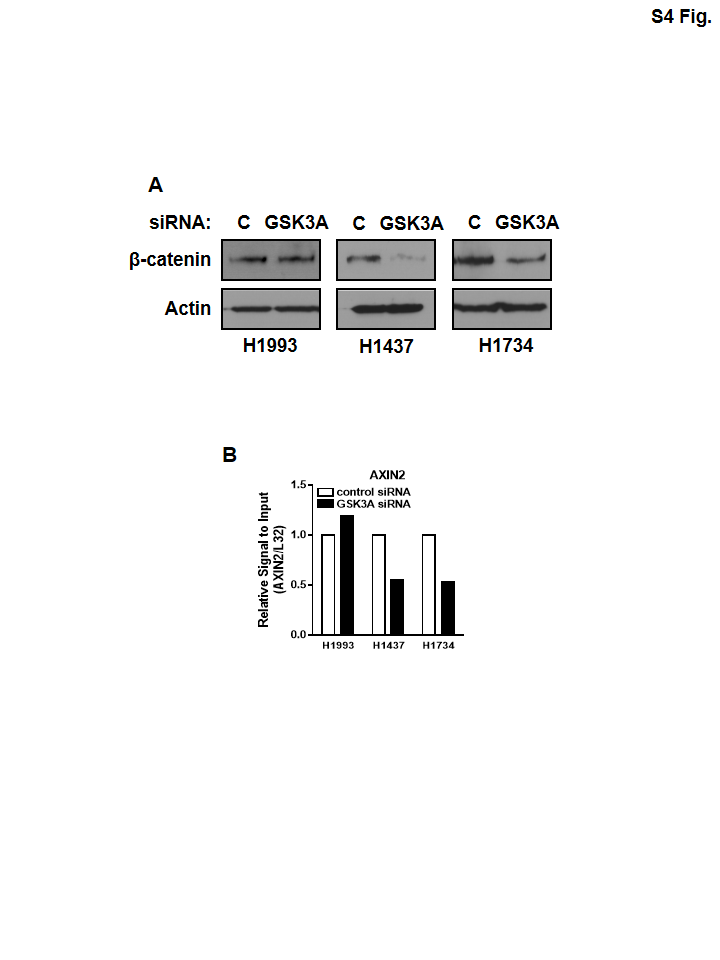

Supplement: S4 Fig — (TIF) [file pone.0153075.s004.tif]

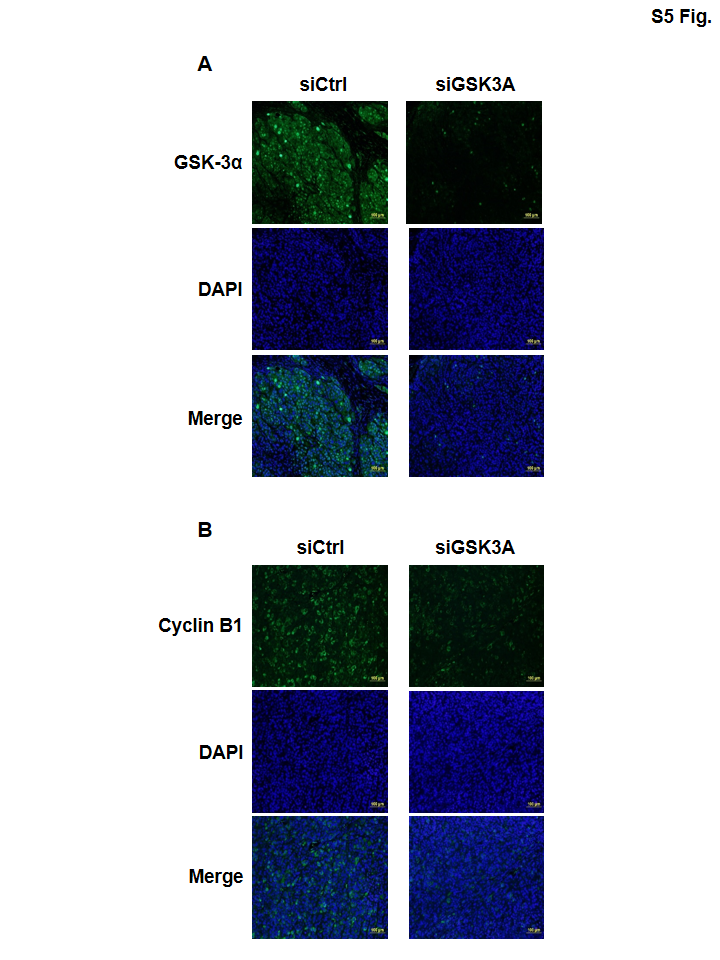

Supplement: S5 Fig — (TIF) [file pone.0153075.s005.tif]
