# Supplementary material for: GSK-3α Is a Novel Target of CREB and CREB-GSK-3α Signaling Participates in Cell Viability in Lung Cancer
Source: PLoS One. 2016 Apr 6;11(4):e0153075. doi: 10.1371/journal.pone.0153075 (PMC4822949; doi:10.1371/journal.pone.0153075)
Supplement: S1 Table — (DOCX) [file pone.0153075.s006.docx]

**SUPPLEMENTARY TABLE**

**S1 Table. Primer sequences for qPCR or ChIP assay and sequences of shRNAs**

| **Primer Name** | **Sequence** |
| --- | --- |
| GSK3A_FW | CAATATTGCAGTGGTCCAGC |
| GSK3A_RV | GGGAACTAGTCGCCATCAAG |
| GSK3B_FW | ACTTCTTGTGGCCTGTCTGG |
| GSK3B_RV | AGCTTTTGGCAGCATGAAAG |
| CREB_FW | TGCAGCTGTAACAGAAGCTGA |
| CREB_RV | AGAGTTACGGTGGGAGCAGA |
| L32_FW | CACCAGTCAGACCGATATGT |
| L32_RV | ACGTTGTGGACCAGGAACT |
| GSK3A_#A_5' ChIP | AGTCGGCTGACAGATTACCC |
| GSK3A_#A _3' ChIP | TCCACAAAAGCCTCAGAGTAGA |
| GSK3A_#B_ 5' ChIP | GTCGAACTCCATTTCCCAGA |
| GSK3A_#B_3' ChIP | TAAGCCCCAGGTGGATACTG |
| GSK3A_#C_5' ChIP | CAGCATGCTTGGAGCTCAT |
| GSK3A_#C_3' ChIP | CCTCAGTGCAGTTGCCACA |
| GSK3A_#D_5' ChIP | CCGCAGTCTCTAACCTTTCG |
| GSK3A_#D_3' ChIP | CAAGCATGCTGGGGAATAGT |
| GSK3A_NS-1_5' ChIP | GCCGACTCAATTTCCCAGAG |
| GSK3A_NS-1_3' ChIP | AGGAAGCAGAGAGCCTGATG |
| GSK3A_NS-2_5' ChIP | CAGTATCCACCTGGGGCTTA |
| GSK3A_NS-2_3' ChIP | CTGTGTATGCCTCCCAGAGC |
| GSK3A_NS-3_5' ChIP | CTGAAAACTGAATGGTGGTTCA |
| GSK3A_NS-3_3' ChIP | GGAGGCTGAAGCAGGAGAAT |
| shScrambled | CCGGCAACAAGATGAAGAGCACCAACTC  GAGTTGGTGCTCTTCATCTTGTTGTTTTT |
| shGSK3A#2 | CCGGGAACAAATCCGAGAGAT |
| shGSK3A#4 | CTACATCTGTTCTCGCTACTA |
| shCREB#1 | ACATTAGCCCAGGTATCTATG |
| shCREB#2 | ACGGTGCCAACTCCAATTTAC |
